# New Model for Weather Stations Integrated to Intelligent Meteorological Forecasts in Brasilia

**DOI:** 10.3390/s25113432

**Published:** 2025-05-29

**Authors:** Thomas Alexandre da Silva, Andre L. M. Serrano, Erick R. C. Figueiredo, Geraldo P. Rocha Filho, Fábio L. L. de Mendonça, Rodolfo I. Meneguette, Vinícius P. Gonçalves

**Affiliations:** 1 Universidade de Brasília (UnB), Campus Darcy Ribeiro, Brasília 70910-900, Brazil; andrelms@unb.br (A.L.M.S.); erick.rollemberg@ieee.org (E.R.C.F.); fabio.mendonca@redes.unb.br (F.L.L.d.M.); vpgvinicius@unb.br (V.P.G.); 2 Universidade Estadual do Sudoeste da Bahia (UESB), Campus Vitória da Conquista, Conquista 45083-900, Brazil; geraldo.rocha@uesb.edu.br; 3 Universidade de São Paulo (USP), Campus São Carlos, São Paulo 05508-030, Brazil; meneguette@icmc.usp.br

**Keywords:** agro-meteorology, IoT, microcontrolled weather station, solar energy, machine learning forecasting, environmental monitoring, cerrado biome, real-time data collection, open-source, open-circuit

## Abstract

This paper presents a new model for low-cost solar-powered Automatic Weather Stations based on the ESP-32 microcontroller, modern sensors, and intelligent forecasts for Brasilia. The proposed system relies on compact, multifunctional sensors and features an open-source firmware project and open-circuit board design. It includes a BME688, AS7331, VEML7700, AS3935 for thermo-hygro-barometry (plus air quality), ultraviolet irradiance, luximetry, and fulminology, besides having a rainfall gauge and an anemometer. Powered by photovoltaic panels and batteries, it operates uninterruptedly under variable weather conditions, with data collected being sent via WiFi to a Web API that adapts the MZDN-HF (Meteorological Zone Delimited Neural Network–Hourly Forecaster) model compilation for Brasilia to produce accurate 24 h multivariate forecasts, which were evaluated through MAE, RMSE, and R^2^ metrics. Installed at the University of Brasilia, it demonstrates robust hardware performance and strong correlation with INMET’s A001 data, suitable for climate monitoring, precision agriculture, and environmental research.

## 1. Introduction

Understanding weather patterns and their changes is essential to optimize agricultural production and ensure food security  [1,2], while being closely related to multiple other sectors such as aviation, general transportation [3], and even influencing local culture [4]. As such, there has been a historical interest in recording various meteorological variables, such as air temperature, relative humidity, atmospheric pressure, rainfall, wind speed, direction, and many others [5]. Traditionally, in conventional weather stations (CWS), measurements used to be taken using analog instruments, such as barographs, anemographs, dry and wet-bulb mercury-based thermometers from thermohygrographs, and many others [5,6,7], with the first AWSs (Automatic Weather Stations) emerging only during World War II, developed by the US Navy [8]. Furthermore, since the 1980s, AWSs have been increasingly used in the agricultural sector due to improvements in the energy efficiency of data loggers and communications [9]. This phenomenon is also due to the advent of low-power and low-cost wireless modules, which motivated their applications to expand from industrial to agricultural applications [10], allowing the development of IoT (Internet of Things) in this field. Given such socioeconomic importance, general academic output to monitor climate changes has increased significantly [11]. Some biomes, such as the Brazilian Cerrado, are known to be getting hotter, drier, and, therefore, more prone to wildfires [12], which highlights the urge for AWSs in such places.

Considering the agricultural applications of IoT, most technical productions in this field occur in a fragmented manner and lack cooperation [13], especially for solutions in early stages of development—thus the importance of open architectures and standards in this matter. Besides all advances, most professional AWS can still cost tens of thousands of dollars [14,15,16], imposing challenges to the large-scale deployment and maintenance of such devices [17], with inexpensive models emerging as a necessity. In this context, real-time meteorological information should be systematically researched, as well as the prediction of renewable energy and estimating the impact of natural disasters [18]. Furthermore, Stith et al. (2018) [19] emphasize the importance of meteorological records for predictive practices based on numerical methods. Such data also make the development of ML (Machine Learning) and DL (Deep Learning) methods viable, as highlighted by Da Silva et al. (2024) [20], Zaytar (2016) [21], and Kreuzer et al. (2020) [22]. In these works, LSTM (Long Short-Term Memory) networks are widely applied to generate accurate hourly forecasts, with the first presenting a public solution applied to the Cerrado region of Brasilia.

Thus, this study aims to provide a complete, open-source, and open-circuit low-cost model for microcontrolled solar-powered AWSs that are capable of being integrated into intelligent forecasts and prove themselves accurate for the Cerrado Region of Brasilia. It is intended to monitor several data, such as temperature, humidity, pressure, UV irradiance, ambient illuminance, anemometry, rainfall, air quality, lightning, and hardware health, as well as estimating total solar irradiance. In addition to being WiFi-integrated, its Web API (Application Programming Interface) is desired to run an MZDN-HF (Meteorological Zone Delimited Neural Network–Hourly Forecaster) [20] compilation to execute intelligent 24 h forecasts and confirm whether or not it is applicable for custom stations in Brasilia without requiring new compilations.

### Related Studies

In the context of microcontroller-based AWS, Parvez et al. (2016) [23] proposes a photovoltaic-powered station based on LM-35, HSM-20G, MPL115A1, GUVA-S12SD, and TEMT6000 modules for temperature, humidity, pressure, UVI (Ultraviolet Index), and illuminance sensing, respectively, along with an LCD (Liquid Crystal Display). In addition to a vast set of air quality sensors, an authorial rainfall gauge and anemometers were proposed. Despite MPL115A1 being discontinued, the prototype is very complete and integrates GSM (Global System for Mobile Communications) communications, focusing on inexpensiveness for popular use in rural areas of Bangladesh. In the next year, Li et al. (2017) [18] developed a GPRS (General Packet Radio Service)-oriented portable station to measure wind speed, wind direction, light intensity, temperature, and humidity in Jinan, China, with a DS18B20 and a DHT11 for thermohygrometry and a BH1750FVI for luximetry. Although it presented good results, with little data loss during transmissions, it should be noted that some of the sensors have a reduced range, as DHT11 varies from 20% to 80% in humidity. Therefore, although prominent, low-cost, and compact, such a prototype might not be suitable for very humid or dry zones. Posteriorly, Megantoro et al. (2021) proposed a wireless ESP-32-based AWS model [24], equipped with a DHT11 and BME280 for thermohygrometry, a rainfall gauge, an anemometer, a UVI module, and several MQ-family sensors for air quality in Airlangga (Indonesia). In addition to its WiFi connection, the author’s model had an LCD for immediate data display. Although not solar-powered, it brought a vast diversity of measurements into a compact prototype with modern sensors and public circuitry details.

Furthermore, Wang et al. (2022) [25] made significant contributions when proposing a solar-powered AWS with open circuit design based on a BME680—a thermo-hygro-barometer plus air quality meter, all in one. That being a more recent work, its sensor choice is both more precise and cheaper than the sets previously reported. BME68X air quality resistance was also paired with and complemented by a ZPH02 dust sensing module. In terms of solar data, the author cites VEML6070 for I^2^C UV irradiance readings, despite its obsoletism [26]. Furthermore, Wang et al. (2022) [25] highlight the social importance of adapting technology to Chinese reality with a national production of weather station models. Regarding air quality, Bhandekar et al. (2024) [27] proposed a solar-powered AWS capable of sensing air quality through a set of many MQ sensors, measuring many gases, vapors, alongside with PM (Particulate Matter) specific modules. Similarly, Fahim et al. (2023) [28] proposed a prototype that monitors air pollutants to infer the AQI (Air Quality Index) through Fuzzy Logic. Although not deployed, nor solar-powered, it presents a significant contribution in the field of A.I. (Artificial Intelligence) integrated AWSs.

Furthermore, in the field of ML-based weather forecasts, an increasing number of research works have been produced, with many relying on LSTMs or Convolutional LSTMs, such as Kreuzer et al. (2020) [22], Hou et al. (2022) [29], Qing and Niu (2018) [30], Ozbek et al. (2022) [31]. In addition, in the Brazilian Cerrado region, Da Silva et al. (2024) [20] propose an encoder–decoder format of LSTM networks, trained and optimized using 5 years of hourly data provided by Station A001 [32], an official INMET (Brazil’s National Institute of Meteorology) AWS located in Brasilia. After some parameter hyper tuning experiments, the model was able to predict a multivariate set of hourly data over a one-day horizon. The model is named MZDN-HL, and after hyperparameter optimization, it achieved a robust performance of 1.32 °C, 7.14%, 0.629 hPa, and 56.7 W/m^2^ of MAE (Mean Absolute Error) for temperature, humidity, pressure, and solar irradiance forecasts, besides being a low-computational-cost solution and easy to implement.

Therefore, the relevance of an AWS that combines wireless integration, solar power, data completeness, open architecture, and good cost–precision balance is clear. Additionally, it is notably interesting to rely on modern sensors that are still available to guarantee the experiment’s full reproducibility and adequate for measurement range, keeping it reliable to local conditions (in this case, the Cerrado biome in Brasilia). Finally, for solutions installed in open environments that are wireless and intended to be minimally energy-saving, the LCD modules’ necessity for in-person data checking, as seen in some works, can be discarded, as well as high-power-consuming sensors. Finally, see Table 1 for comparisons between this paper’s contributions and some prominent works in the field.

## 2. Materials and Methods

**Figure 1:**
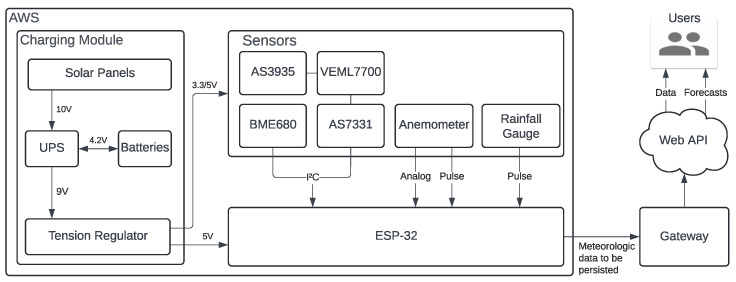
General AWS diagram.

The proposed AWS contains a set of electronic and electromechanical sensors integrated into a central board that, being powered by batteries and solar panels, connects its inputs to an ESP32 WROOM-32D microcontroller, designed and distributed by Espressif, in Shanghai, China. This one is responsible for processing the input signals and serializing the data for WiFi transmission to a Web API that persists, displays, and produces MZDN-HF based forecasts. See Figure 1 for structural clarification.

### 2.1. Sensors

**Figure 2:**
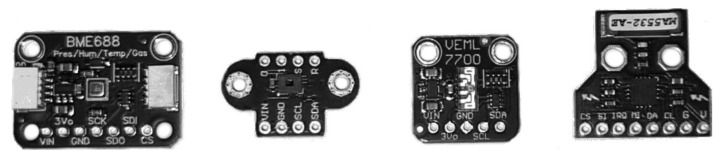
BME688, AS7331, VEML7700, and AS3935, respectively.

As for the electronic sensors, the chosen set was BME688, AS7331, VEML7700, and AS3935 for thermo-hygro-barometry (plus air quality resistance), ultraviolet irradiance (UVA, UVB, and UVC separately), luximetry, and lightning, in this respective order. See Figure 2 for better visualization of the modules. The BME688 was specifically chosen due to its multisensing capabilities, bringing it simplicity and reliable information over four important weather variables. Its air quality module is MOX (Metal Oxide)-based, that is, it relies on a heated metal whose resistance varies through exposition to reducing gases, such as CO, $H_{2}$ , ${\mathrm{NH}}_{3}$ , $H_{2}S$ , VOCs (Volatile Organic Compounds), etc. Although BME688 air quality resistance is not directly convertible to any specific pollutant concentration in PPM or PPB, it still offers good relative reports over time. AS7331 was chosen for being on of the few market solutions up to the current date to actually output results in irradiance (W/m^2^) for three UV spectrums; VEML7700 due to its high maximum value of 120 kLx in comparison to other modular lux-meters; and AS3935 due to being one of the few lightning sensors available in the market at this moment. All four sensors were chosen considering their small size, electrical efficiency, and ease of implementation, as evidenced by their I^2^C compatibility, allowing their data buses to be shared.

As electromechanical sensors, a generic tipping bucket rain gauge and anemometer with wind vane are included in the proposed solution—all being hall-effect based with magnets attached to rotating pieces. Both a pluviometer and an anemometer can be built from scratch via 3D printing or bought from local providers. In this experiment, they are, respectively, PB10 and SVDV10, provided by Usinainfo [33], chosen due to it being a national Brazilian producer.

**Figure 3:**
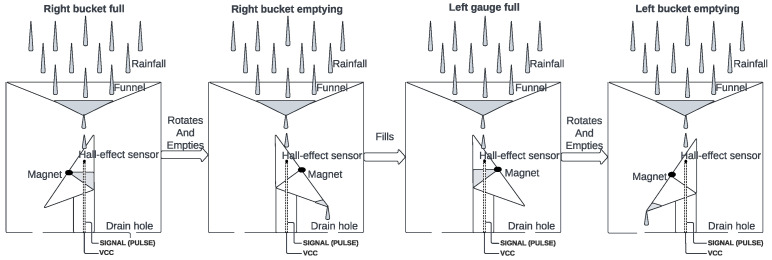
A tipping bucket rain gauge performing its cycle.

For any generic tipping bucket pluviometer, as in Figure 3, an event occurs when one of its two buckets is full and, being heavy enough, it rotates, spilling its water and forcing the opposite bucket up, towards the upper funnel. When this other bucket is filled, its weight forces another rotating movement that empties it, completing the cycle with the first bucket back upwards. With a small magnet in the bucket’s structure and a hall-effect sensor facing it, each movement is sensed as a pulse and accounts for a certain volume of water. In this current experiment, the chosen capacity was that of 0.25 mm. This whole process allows the measurement of a cumulative value over a given interval.

**Figure 4:**
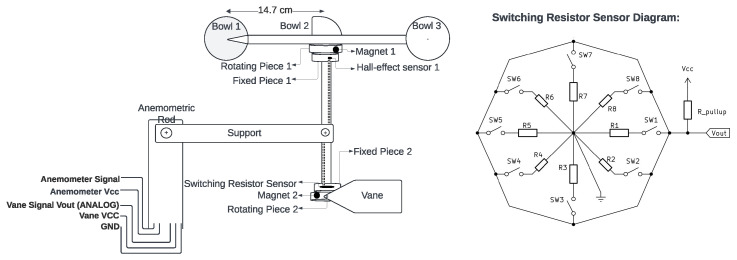
An anemometer with a wind vane (**left**) and the vane rotation-magnet-based resistor switching circuit (**right**).

For the anemometer, portrayed in Figure 4, the principle is similar. Each one of its rotations triggers a hall sensor with a magnet, switching two wires and accounting for one pulse on the microcontroller. The amount of pulses received is then used to calculate the average tangential speed during Δt , with the radius between the axis and the cups being a mechanical constant of the system. Its wind vane is also hall-effect-based, relying on a magnet switching over eight different resistors to influence the final tension value. Finally, see Table 2 for technical details on both electronic and electromechanicals.

### 2.2. Solar Powering

**Figure 5:**
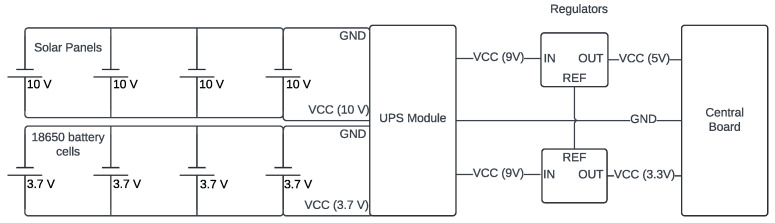
Schematic diagram of the charging and power supply module.

Finally, autonomy is a key factor for IoT projects in rural areas. Therefore, long-lasting batteries are required to keep the station running for 12 h without sunlight and panels capable of fully recharging them during daylight hours, even on cloudy days. The 12 h value was chosen considering medium and low latitudes (consistent with the Brazilian reality), where the day and night ratio is about 1:1, even in the solstices. To achieve this autonomy, 4 cells of 2600 mAh and 3.7 V 18650 were used, along with 4 panels of 3.5 W/10 V and a 5 V–12 V to 9 V UPS charging module. See Figure 5.

The panels are connected in parallel to maintain a fixed nominal voltage of 10 V and quadruple the current. The input voltage limit of the DC UPS module is not exceeded, and a higher charging speed is obtained for the four battery cells (also connected in parallel to keep the tension and quadruple capacity). The 9 V output is subsequently regulated to 5 V and 3.3 V to power the main board. Finally, a total battery capacity of 10,400 mAh is achieved. That is enough to keep a 500 to 650 mA average consumption circuit for approximately 16 to 21 h until fully discharged (above the intended 12 h).

### 2.3. Components Costs

As a differential for the proposed solution is the establishment of an open framework integrating inexpensive sensors and allowing for its overall minimal cost, average market prices must be transparently shown. Therefore, see Table 3 for referenced average values up to 2025-05-16, with its first part being restrictive to electronics and the second to structural pieces. The informed values in both tables do not include the labor force to assemble and solder any of the components. Values may also vary with time and place, being susceptible to transportation and importation taxes.

### 2.4. Firmware

The firmware for the proposed application must be capable of collecting data from the sensors mentioned in Section 2, processing them, and sending them over WiFi to a web API responsible for storing the received data. In general, it is essential to be robust, energy efficient and capable of handling user input to configure the WiFi connection.

To this end, the first pseudocode (Algorithm 1) is responsible for initializing the ESP-32 WDT (Watchdog Timer) to recover from eventual unexpected crashes, disabling unused modules, reducing the clock frequency to 80 MHz (for battery saving), and enabling the WiFi module only to sync a clock variable with an external NTP server before disabling it again (necessary for future data packages to keep a date and time reference). Anemometry and rainfall threads are also initialized and will be triggered asynchronously by external interrupts from electromechanicals on their pins. Algorithm 2 presents these threads, each increasing its global variables ( _an or _plv , both represented as _eventCounter ) by checking a minimum 25 μs debouncing interval since the last event.    
**Algorithm 1:** Setup code.
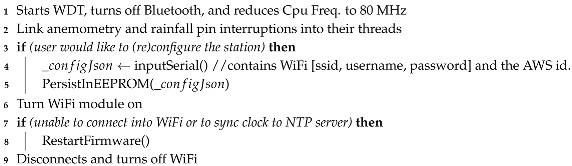


**Algorithm 2:** Thread function model.

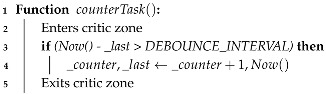



Finally, Algorithm 3 contains the main loop, performing data collection followed by a 60 s watchful light–sleep (Algorithm 4), which is interrupted by external events to count them before returning to sleep until the stipulated time elapses. After 10 min, accumulators and counters are used to calculate the average or total of each measured feature. Then, the data are serialized to JSON and sent via HTTP to the Web API. After packages are sent, variables are reset after and sensors are health-checked and restarted if necessary. Additionally, to avoid noise during analog reads (battery and wind direction), a low-pass filter (with a smoothing factor of α=0.2 ) is applied over 200 samples, followed by a median (Algorithm 5).
**Algorithm 3:** Loop with sensor data collection.
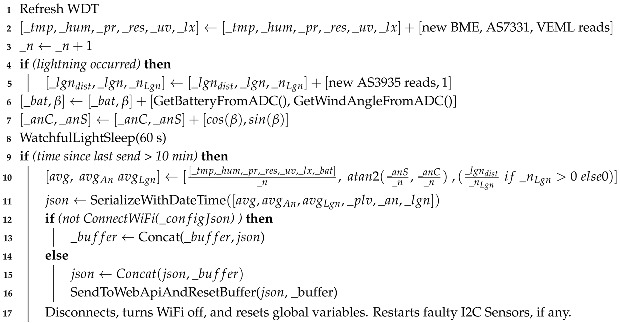


**Algorithm 4:** Light–sleep, watchful to interruptions.

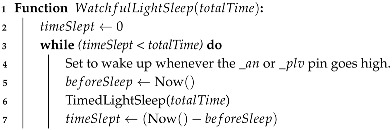



**Algorithm 5:** Low-pass median filter.

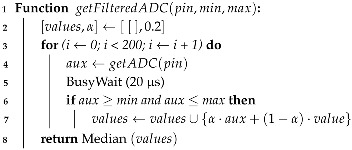



### 2.5. The MZDN-HF Model for Brasilia

**Figure 6:**
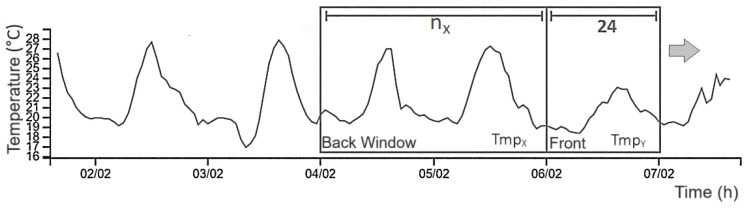
Sliding window $Tmp_{X}\in X_{i},Tmp_{Y}\in Y_{i}$ . Adapted from Da Silva et al. (2024) [20].

The MZDN-HF model approximates a windowed output $Y_{i}$ , containing submatrices for temperature ( Tmp ), humidity ( Hr ), pressure ( Pr ), solar irradiance ( Rd ), and rainfall ( Plv ) by a ${\hat{Y}}_{i}$ value calculated trough a transformation T{·} . Such transformation considers an input window sample $X_{i}$ with the same submatrices, except for the rainfall, and uses a set of optimized hyperparameters $\hat{h}$ to minimize the error E(·) . In this context, $h_{f}$ denote all the fixed hyperparameters and *h* the variable ones to be hypertuned. See the equations in Expression (1) formalizing it. Finally, the process T{·} basically runs an LSTM encoder–decoder model from $X_{i}$ to $Y_{i}$ . See Figure 6 for better comprehension of data windowing.

(1) $$\begin{array}{c} X_{i},Y_{i}=\left[\begin{array}{ccccc} Tmp_{X}^{1} & Hr_{X}^{1} & Pr_{X}^{1} & Rd_{X}^{1} & Plv_{X}^{1}\\ \vdots & \vdots & \vdots & \vdots & \vdots \\ Tmp_{X}^{n_{X}} & Hr_{X}^{n_{X}} & Pr_{X}^{n_{X}} & Rd_{X}^{n_{X}} & Plv_{X}^{n_{X}} \end{array}\right],\;\left[\begin{array}{cccc} Tmp_{Y}^{1} & Hr_{Y}^{1} & Pr_{Y}^{1} & Rd_{Y}^{1}\\ \vdots & \vdots & \vdots & \vdots \\ Tmp_{Y}^{24} & Hr_{Y}^{24} & Pr_{Y}^{24} & Rd_{Y}^{24} \end{array}\right]\\ \hat{h}={\mathrm{argmin}}_{h}\left(E(Y,\hat{Y})\right)\;|\;\hat{Y}=T\left\{X,h,h_{f}\right\} \end{array}$$

**Figure 7:**
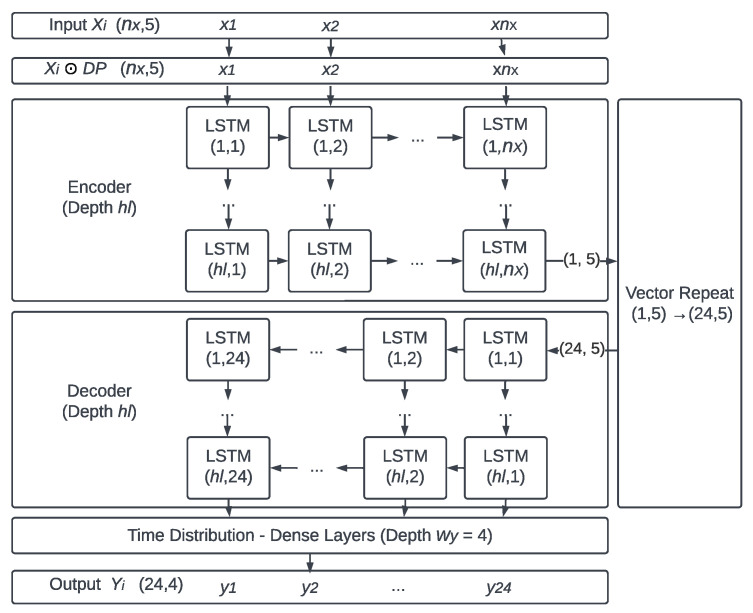
DP , $n_{X}$ , hl represent some of the hyperparameters: Dropout matrix, input window length, and hidden layers number. Adapted from Da Silva et al. (2024) [20].

When analyzing the inner architecture for the T{·} process (Figure 7), the amount of input features $w_{X}=5$ , output features $w_{Y}=4$ , and the output sequence length $n_{Y}=24$ must be comprehended as initial scope parameters. Once input is made, the first LSTM encodes the entry window $X_{i}$ of variable order ( $n_{X}$ , 5) in a fixed-size vector (1, 5), being subsequently repeated 24 times to generate a two-dimensional matrix (24, 5). This is then decoded in the subsequent LSTM network and processed by a densely connected network. Its output, now of order (24, 4), comprises a response matrix $Y_{i}$ . It can be seen, therefore, that the presented arrangement allowed transforming the dynamic sequence ( $n_{X}$ , 5) into a response of fixed size (24, 4), as desired for the defined scope of a day-long hourly forecast.

### 2.6. Data Adaptations to Fit MZDN-HF

The MZDN-HF model has a known compilation [34] for A001 data [32] and can be applied to adjacent locations by applying barometric corrections. Since A001 is one of INMET’s official AWS in Brasilia and counts with reliable temperature, humidity, pressure, dew point, wind speed, direction, solar irradiance, and rainfall, it is a good source of comparison for meteorological data collected in this region. Except for the dew point, all of its measurements can be compared and correlated with those gathered by the aimed sensors in Section 2.1. According to Da Silva et al. (2024) [20], the A001 dataset has shown optimal performance in MZDN-HF with $\hat{h_{1}}=\left\{hl=10,p_{dpout}=0,n_{X}=24,bid=1\right\}$ , meaning 10 hidden layers, no dropout, 24 back-steps at each window, and bidirectional LSTMs.

Regarding barometric corrections, the pressure P2 in a given point of altitude h1 can be easily estimated based on the pressure P1 of another location near enough at the altitude h2 . Based on Wallace (2006) [35], and having $T_{0}$ as the standard average temperature in the sea level, *L* as the decay of temperature, *g* the gravity, *M* the air molar mass, and *R* the ideal gases constant, the Equation (2) is proposed as the milestone for barometric conversions. A few values can be assumed in order to operate it, such as *R* = 8.3144598 J/(mol·K), *M* = 0.0289644 kg/mol, *g* = 9.8 m/s^2^, $T_{0}$ = 288.15 K, and *L* = 0.0065 K/m.

(2) $$P_{2}=P_{1}\times {\left(\frac{T_{0}-L\cdot h_{2}}{T_{0}-L\cdot h_{1}}\right)}^{\frac{g\cdot M}{R\cdot L}}$$

Furthermore, considering that MZDN-HF uses the solar irradiance from the complete irradiated spectrum ( $Rd_{total}$ ), it is necessary to approximate it based on the UV irradiance ( $Rd_{UV})$ measured by the AS7331 as a sum of UVA, B, and C channels. It is known that UV composes about 8% of the total spectrum for direct incidence at the top of the atmosphere [36]. Yet for the zenith case, slightly lower values are implied at the ground level, varying with altitude, aerosol, ozone concentrations, etc. For a more general case, at the ground level for any solar incidence angle *z*, a broad conversion is possible by calculating the air mass factor *m* and applying it along with the attenuation coefficient $\tau_{uv}$ (for the UV spectrum) in Equation (3). This model is derived from Gueymard’s (2003) [37] contributions and the Beer–Lambert Law, which establishes irradiation exponential decay on atmosphere.

(3) $$\begin{array}{cc} m & =\frac{1}{\cos\left(Z\right)+0.50572\cdot {\left(96.07995-Z\right)}^{-1.6364}}\;\forall \;0\leq Z\leq \frac{\pi }{2}\\ Rd_{\mathrm{total}} & =\frac{Rd_{UV}}{r\cdot e^{{-\tau }_{uv}\cdot \left(m-1\right)}},\;\mathrm{where}\;r≲0.08\;\mathrm{and}\;\tau_{uv}\in \;]0.3,0.5[ \end{array}$$

As already mentioned, the MZDN-HF intended compilation is A001-based, being related to Brasilia’s climatology, as well as the prototype here proposed. Although the meteorological behavior between the points should be overall similar, microclimatic differences might emerge (besides the well-known barometric issue and irradiance conversions), such as small temperature and/or humidity offsets. In order to mitigate the forecast error when comparing a prototyped AWS’s data to its own-based forecasts, Da Silva et al. (2024) [20] emphasize the need for microclimatic transformations between A001 and custom sources, as long as they are in the same biome and therefore share the same climatological properties. Such transformations would bring collected data to the A001 domain before inputting them into the compilation and then transforming the forecasted output back to the collected domain. If differences are small and linear, simple first-order regressions can be used, as well as their inverse functions. However, if the observed discrepancies are too high, one must consider instrumental errors or new compilation and parameter hypertuning for the custom dataset.

### 2.7. Implementation

**Figure 8:**
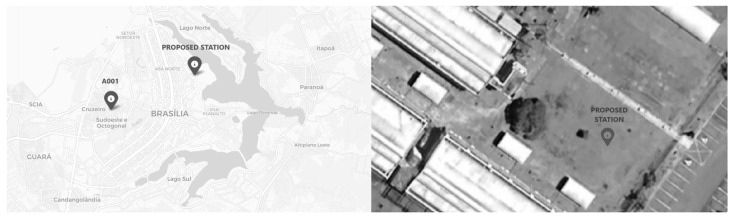
A map with A001 and the proposed AWS locations plus an aerial view from the second one.

The developed prototype—abbreviated to PRT—was installed at UnB, in the Institute of Biology (IB), at 1030 m altitude and at the latitude and longitude coordinates (−15.767210, −47.864540). Approximately 7 km from it, A001 lies at (−15.791222, −47.925200) 1161 m elevation, in INMET. The distance is expected to cause a small climate divergence, as well as UnB being slightly warmer due to higher urbanization and lower altitude. See Figure 8.

**Figure 9:**
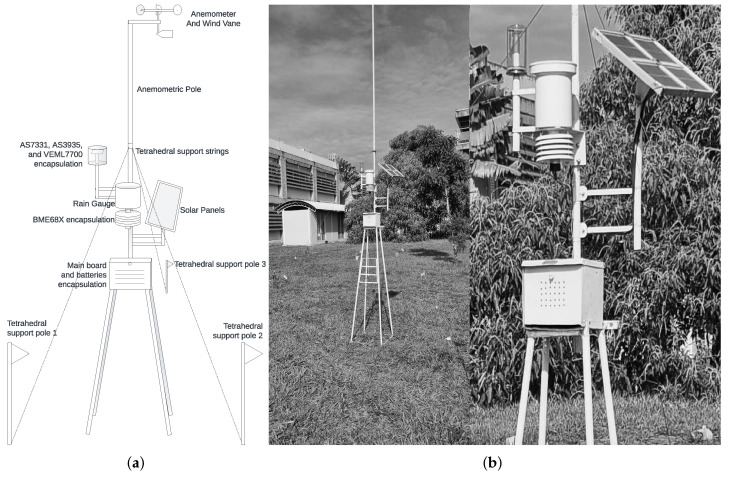
Technical diagram with the proposed station physical body (**a**). Proposed station at UnB (**b**).

**Figure 10:**
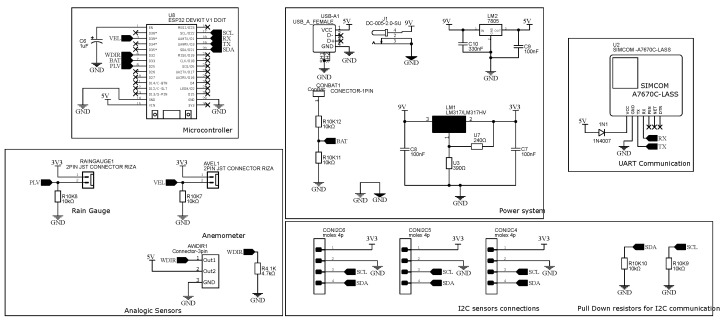
Circuit diagram. 4G SIM module and SCD41 CO_2_ sensor listed for future compatibility. * Input only pins.

Furthermore, Figure 9 clarifies the physical implementation and its surroundings. The central box contains the microcontroller (placed on the main board), along with the batteries. Right over it, BME688’s encapsulation positioning lies 2 m high, like INMET’s recommendations for thermic monitoring, and the pluviometer was placed just above it. On the left side and slightly higher, the AS7331, VEML7700, and AS3935 encapsulations were made with transparent glass to allow solar light reception, also avoiding metal usage to prevent magnetic isolation on the lightning sensor. The location is wide open, ventilated, and far from objects that could shadow the UV and visible light sensors. The solar panels were installed on the opposite side to prevent occlusion and also receive the proper light they need. Finally, the anemometer pole is raised to 4.5 m high and three strings tense the structure tetrahedrally. The microcontroller runs the firmware explained in Section 2.4, compiled and programmed with the Arduino IDE. The public repository for this research [38] contains further details on the firmware. See the circuit design in Figure 10.

**Figure 11:**
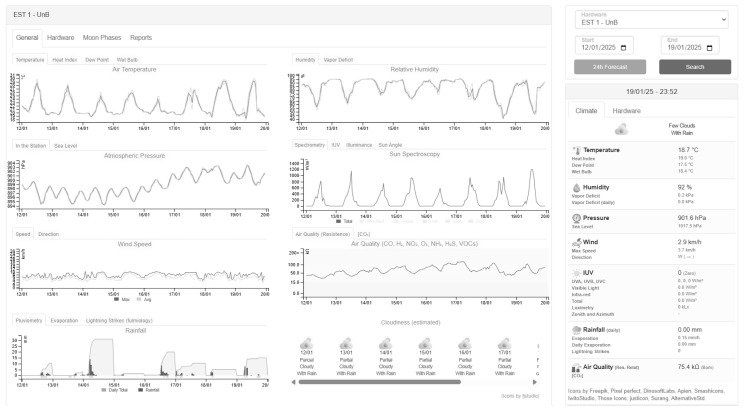
Web interface developed displaying hourly data.

Finally, the Web API responsible for receiving and displaying the data was developed with the Django Framework 4.0.0 and stores data in a PostgreSQL 14.0 database. Although the database was arbitrary, with pretty much any being viable, the back-end was chosen to be developed in Python 3.0 in order to better integrate with the MZDN-HF public library available in Da Silva et al.’s work (2024) [20], which runs in this same language. Still, for compatibility reasons, the Tensorflow 2.0 version was imported to run it. Based on the firmware 10 min base packages, the API endpoints operate hourly arithmetic averages for most time series, with only rainfall and lightning being an hourly sum of their occurrences. Finally, its front-end is browser-based, built with HTML5, CSS3, and JavaScript 1.5, with the help of VueJS 2.7.7 for web components and C3 Chart 0.7.15 for interactive graphs (see it in Figure 11).

### 2.8. Data Analysis

When verifying weather data quality, a good approach is to compare time series from the evaluated AWS and one official station, which serves as a credible reference for the selected period. The sampled data, in order to ensure robustness, is expected to be that of at least three weeks on hourly data, totalizing 504 elements in each series. During the comparison, statistics such as MAE, RMSE (Root Mean Squared Error), and $R^{2}$ (Coefficient of Determination) are applied over the chosen data sample to acquire the absolute error, the root of the standard deviation, and the correlation coefficient between the sets. See Equation (4) for those metrics. In these, *Y* stands for the INMET’s A001 reference data and $\hat{Y}$ the prototyped station series. It should be noted that the conversion of barometry via Equation (2), due to altitude differences, and the estimation of $Rd_{total}$ parameterized by $Rd_{UV}$ through Equation (3) to guarantee data compatibility.

(4) $$\begin{array}{c} MAE\left(Y,\hat{Y}\right)=\frac{1}{n}\sum_{i=1}^{n}|Y_{i}-\hat{Y_{i}}|\\ MSE\left(Y,\hat{Y}\right)=\frac{1}{n}\sum_{i=1}^{n}(Y_{i}-\hat{Y_{i}}{)}^{2}\\ RMSE\left(Y,\hat{Y}\right)=\sqrt{MSE(Y,\hat{Y})}\\ R^{2}\left(Y,\hat{Y}\right)=1-\frac{\sum {\left(Y_{i}-\hat{Y_{i}}\right)}^{2}}{\sum {\left(Y_{i}-\bar{Y}\right)}^{2}} \end{array}$$

Similarly, when forecasts are tested, these same statistics can be applied to compare the events that occurred in the prototyped station (now *Y*, with respect to prognostics) and what was forecasted ( $\hat{Y}$ , in this context) via the chosen model (MZDN-HF). Since the model produces 24 h forecasts, a sequence of daily concatenations can be produced referenced at midnight and then compared with the truly occurred data.

## 3. Results

### 3.1. Data Registered

Data were collected from 2025-01-12 to 2025-02-01 [39], a three-week hourly interval that contained episodes of bright sun and heavy rain in Brasilia. With these records, it is possible to analyze both hardware performance, meteorological information correlated with A001, and MZDN-HF performance on custom data.

#### 3.1.1. Hardware Performance

**Figure 12:**
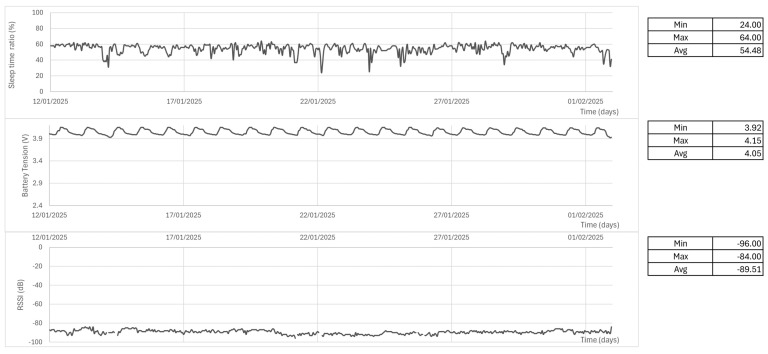
Performance data: MCU sleep ratio, battery voltage, and RSSI.

During the analysis period, the prototype station performed well, was fully operational, and had a minimum battery level at 3.92 V, only 0.28 V discharged from its maximum (4.2 V). In addition, ESP32 was kept in light sleep for about 54.5% of the time and, although the WiFi signal occasionally went off, the readings were buffered in flash memory until the network was back on. The longest period of data buffering due to poor signal was that of 3 h in 2025-01-14. See Figure 12. RSSI was overall very poor, averaging −89.5 dB, but was already enough for the prototype to keep connection most of the time.

#### 3.1.2. Meteorological Data

**Figure 13:**
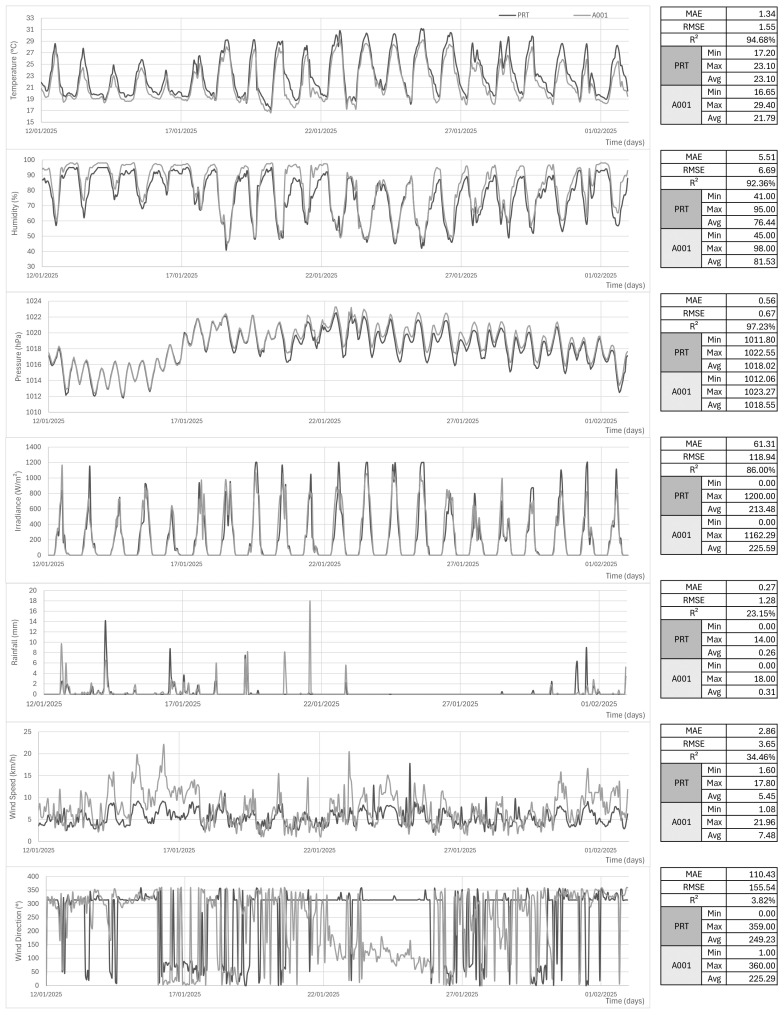
PRT and A001 data represented in dark and light gray. Statistic boards on the side. Series are temperature, humidity, pressure, irradiance, rainfall, wind speed, and direction from top to down.

In this same period, the temperature, humidity, sea-level pressure (reduced by Equation (2)), and total irradiance (calculated by Equation (3) hyperparameters r=0.07 and $\tau_{uv}=0.4$ ) performed well compared with A001, with a $R^{2}$ of 95.68%, 92.36%, 97.23% and 86%, with MAEs of 1.34 °C, 5.51%, 0.56 hPa and 61.31 W/m^2^, respectively. Despite the geographic distance, a satisfactory high similarity is noticed on these series. On the other hand, the average wind speed showed a lower $R^{2}$ of 34.46% and MAE of 2.86 km/h, followed by rainfall, with 23.15% and 0.27 mm. Finally, the wind direction represented the weakest $R^{2}$ , 3.82%, and a MAE of 110.43°. Analyzing the averages, the prototyped station (PRT) was 1.31 °C warmer (as expected, due to lower altitude), 5% drier, had 0.53 hPa lower pressure, 12.1 W/m^2^ less solar irradiance, 0.04 mm less rain, 2.03 km/h less wind speed, and a 23.94° drift in the average direction. See Figure 13.

**Figure 14:**
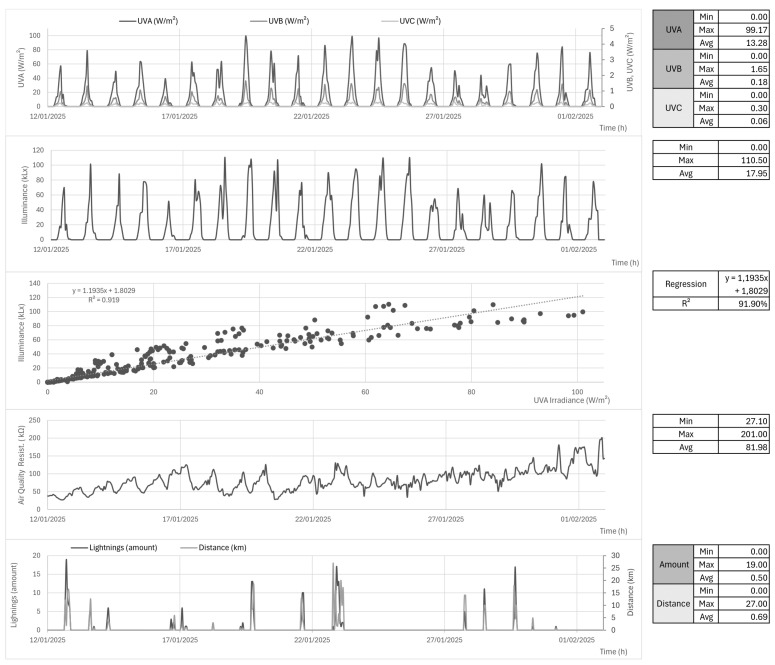
Data exclusive to PRT. Luximetry, UVA/B/C irradiance, air quality resistance, UVA-lux correlation, and lightning, respectively, from top to down.

Finally, it should be noted that some of the proposed AWS variables are not sensed by A001 and were analyzed separately. That is the case of illuminance and UVA/B/C irradiance, air quality resistance, lightning, and lightning average distance. Note that the BME688 resistance base level can vary from each module batch; thus, it must be analyzed relatively over time. Their series is shown in Figure 14, along with the UVA–luximetry correlation, which exhibited an $R^{2}$ of 91.9%. Luximetry ranged from 0 to 110 kLx, UVA from 0 to 99.17 W/m^2^, UVB from 0 to 1.65 W/m^2^, UVC from 0 to 0.3 W/m^2^, gas resistance from 27 kΩ to 201 kΩ, and lightning from 0 to 19 (hourly), with distances from 1 to 27 km.

### 3.2. MZDN-HF Adaptation Performance

**Figure 15:**
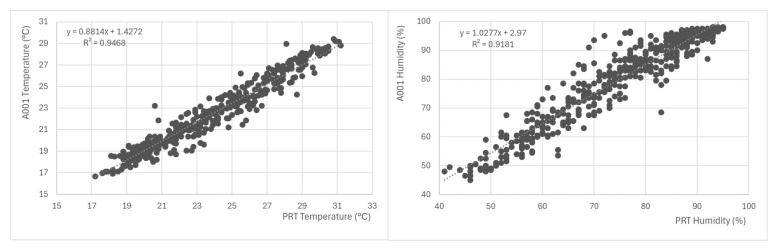
PRT to A001 Linear Correlation and Regression. Temperature (**left**) and humidity (**right**).

**Figure 16:**
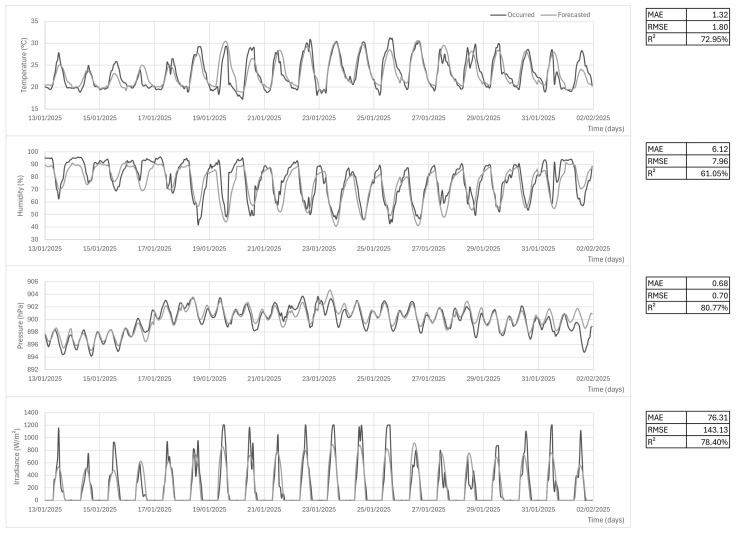
PRT data and MZDN-HF forecasts (temperature, humidity, pressure, and solar irradiance from top to bottom). MAE, RMSE, $R^{2}$ on the right.

When integrating the MZDN-HF known compilation (trained with the A001 dataset) into the proposed station, linear regressions were performed to approximate the A001 temperature and humidity domain based on PRT to preserve compatibility when inputting *X*. The *Y* results were reversed back to PRT domain using the same regressions (See Figure 15). The pressure was converted, more precisely, through Equation (2), between the two points, and total irradiance was understood as uniform at both stations once Equation (3) is applied. Hourly 24 h forecasts were generated at midnight of each day and joined to form the [2025-01-13, 2025-02-02] interval. The forecasted time series was, then, compared with the occurred data in the proposed AWS, as seen in Figure 16. The MAE, RMSE, and $R^{2}$ metrics were 1.32 °C, 1.80 °C, and 72.95% for temperature; 6.12%, 7.96% and 61.05% for humidity; 0.68 hPa, 0.70 hPa and 80.77% for pressure; and 76.31 W/m^2^, 143.13 W/m^2^, and 78.4% for solar irradiance. The latter appears to have failed to properly capture the daily seasonality curve in the predictions, leading to poor performance compared with the good results exhibited by the others. The final performance report can also be found separately in Table 4.

## 4. Discussion

The collected data have shown good correlation with A001, despite their geographical distance and altitude differences, with temperature, humidity, sea-level pressure, and irradiance performing $R^{2}$ values above 90% for the first three and 85% for the last one. Their MAE, MSE, and average differences were also reasonably low. Finally, rainfall, wind speed, and wind direction had lower correlations and slightly higher errors, which is expected since rain occurs sparsely and the A001 pole is 10 m long (more than twice the length of this prototype). Although a higher pole could be prototyped, PRT would lose in simplicity both in installation and supporting structure. Although rain may be localized in Brasilia, it is worth noting that the time series average of PRT and A001 pluviometry diverged only by 0.04 mm, very little in this variable context.

In general, the quality of the meteorological data presented is satisfactorily high. Data completeness is also a differential, as the referred station counts with UVA/B/C, illuminance, air quality, and lightning monitoring. The UVA–luximetry correlation was that of $R^{2}$ = 91.9%, also pretty high and accounting for cross-validation purposes. In addition, most lightning was registered close to (when not during) rainy periods, which also corroborates this measurement accuracy. However, it must be noted how close the luximetry peak (110 kLx) was to the sensor maximum range (120 kLx), raising questions about high illuminance measurements reaching a plateau near the sensor maximum on brighter days in the future. In this context, VEML7700 could be protected with a partial light-blocking dome to ensure sensed values would always be lower than maximum and, then, compensated for in software to match a precision lux meter—therefore allowing final results to exceed 120 kLx cap value and preventing information loss on peaks. The air quality resistance values of BME688 were higher during the night, possibly due to the vegetal and anthropogenic liberation of VOCs during daytime. It must be noted that BME688 is also sensitive to smoke; therefore, it is important in the Cerrado biome context, which suffers from natural seasonal and anthropogenic wildfires. Such events are expected to significantly lower the resistance output, although they did not occur in the observed time window.

Additionally, Section 3.1.1 achieved a good 54% light–sleep ratio, healthy battery voltage, and no data loss (due to flash memory buffering when RSSI was poor), confirming its robust operation.

Regarding MZDN application, the temperature, humidity, and pressure RMSE (1.80 °C, 7.96%, 0.70 hPa) revealed satisfactory performance, close to the results presented by Da Silva et al. (2024) [20] (1.77 °C, 9.65%, 0.82 hPa). However, irradiance did not perform as well, exhibiting an RMSE of 143.13 W/m^2^, about 22% higher than the 117 W/m^2^ archived by the mentioned author in the A001 database, possibly due to different PRT-A001 sensor responsiveness (specially in high zenithal angles) combined with inaccuracies inherent in the approximation made by Equation (3). When analyzing each feature separately and searching for studies in different datasets, results were revealed to also be close to those of Kreuzer et al. (2020) [22], Hou et al. [29], Qing and Niu (2018) [30], and Ozbek et al. (2022) [31], which include some of the cutting-edge works in the field of hourly weather forecasting on a 1-day horizon. See Table 5.

Further enhancements are possible in data gathering, both with new sensors whenever further versions are released and with adding new electronic modules for more variables being monitored, like dust or specific gas concentrations, sky images, etc. In addition, the board contains an unused UART bus which, in the schematic, was meant to be integrated with an A7670C 4G module as a WiFi fallback. The designed board also has a socket for an SCD41 sensor, which is a carbon dioxide concentration I^2^C module. Such a sensor can pair well with the relative air quality resistance of BME688 in future studies. These enhancements are likely to expand the environmental comprehension of urban areas with high pollution levels.

While the system performed well in Brasilia’s Cerrado biome, its accuracy in other regions with different climatic conditions, such as tropical rainforests or arid zones, remains to be validated. Future reproductions of this study could deploy the system in diverse environments to evaluate its generalizability. In this juncture, new stations could be reproduced to expand climate monitoring in Brazilian territory, especially considering the low-cost components on which the design is based. For this purpose, the firmware code and the board schematics were left open for public use. With respect to forecasts, new MZDN-HF compilations can be adapted and applied, as well as expanded and combined with other models to enhance accuracy and cover more variables, such as rainfall, wind speed, direction, etc.

## 5. Conclusions

The prototype developed demonstrated the feasibility and reliability of a low-cost (about a few hundred USD), open-source, open-circuit, and AI-integrated AWS, meeting all defined objectives. Using modern sensors and photovoltaic power, the system operated autonomously with stable performance and without data loss, even under adverse network conditions.

Meteorological data collected over a three-week period presented strong $R^{2}$ correlation with the A001-INMET reference station in most metrics, particularly for temperature, humidity, pressure, and irradiance. The MZDN-HF integration enabled accurate 24 h forecasts using data collected in real time by the prototype, without requiring retraining or local recompilation. The MAE and R^2^ values obtained demonstrate the potential of this integrated approach for practical use in environmental and agro-meteorological contexts.

Furthermore, the proposed system was developed entirely with personal funding from the authors, focusing on its affordability and reproducibility. All hardware schematics, firmware, and collected data have been made openly available to facilitate scientific cooperation and the dissemination of accessible meteorological infrastructure in regions with limited resources.

Thus, because of its low cost and disclosed nature, it represents a step forward in democratizing access to automated weather stations and helping to center technology development toward popular needs. Therefore, it facilitates agricultural and academic production in countries in the third world, such as Brazil, benefiting its peasantry and meteorological researchers through the exchange of technology and mutual collaboration. As a direct consequence, it also represents a step forward in the field of monitoring climatic changes, especially in high-risk biomes such as the Cerrado region.

## Figures and Tables

**Table 1:** Comparison with similar works.

Feature	Parvez et al.	H. Li et al.	Megantoro et al.	Wang et al.	Fahim et al.	Bhandekar et al.	This Study
	**(2016) [23]**	**(2017) [29]**	**(2021) [24]**	**(2022) [25]**	**(2023) [28]**	**(2024) [27]**	**(2025)**
Temperature and Humidity	✔	✔	✔	✔	✔	✔	✔
Pressure	✔		✔	✔	✔		✔
UV Irradiance/UVI	✔		✔	✔			✔
Illuminance	✔	✔	✔				✔
Rainfall	✔		✔				✔
Wind Speed and Direction	✔	✔	✔				✔
Air Quality	✔		✔	✔	✔	✔	✔
Lightning							✔
Solar Powered	✔	✔		✔		✔	✔
Wireless Connection	✔	✔	✔	✔	✔	✔	✔
Performs Forecasts							✔
AI Integrated					✔		✔
Comp. to National AWSs							✔
Hardware Monitoring		✔					✔
Public Circuit Design	✔		✔	✔	✔		✔
Public Firmware							✔

**Table 2:** Used sensors descriptions. * Plv∈[0.25,50] , ** Vel∈[0.9,80] .

Sensor	Measurement	Symbol	Output	Protocol	Error Margin	Unit
BME688	Temperature	Tmp	Digital	$I^{2}C$	±0.5	°C
BME688	Relative Humidity	Hum	Digital	$I^{2}C$	±3.0	%
BME688	Pressure	Pr	Digital	$I^{2}C$	±0.6	hPa
BME688	Air Quality Resistance	Res	Digital	$I^{2}C$	-	kΩ
AS7331	UV Irradiance	$Rd_{uv}$	Digital	$I^{2}C$	-	mW/cm^2^
VEML7700	Illuminance	Lx	Digital	$I^{2}C$	-	Lx
AS3935	Lightnings	Lgn	Digital	$I^{2}C$	-	-
AS3935	Lightnings Distance	$Lgn_{dist}$	Digital	$I^{2}C$	-	km
PB10	Pluviometry	Plv	Digital	-	±1.2–2.5 *	mm/h
SVDV10	Anemometry	An	Digital	-	±0.9–4.0 **	km/h
SVDV10	Wind Direction	Dir	Analog	-	-	°

**Table 3:** Estimated prices based on average market values as of 2025.

Component	Price (USD)	Amount	Total (USD)
10 W 12 V Solar Panel	7.00	4	28.00
DC 5 V 9 V UPS Module	1.80	1	1.80
18650, 2600 mAh Battery Cells	2.30	4	9.20
ESP-32 WROOM 32D	4.20	1	4.20
BME688	20.00	1	20.00
AS7331	16.00	1	16.00
VEML7700	5.00	1	5.00
AS3935	24.00	1	24.00
1″ SAE Steel Industrial Round Tube	10.00/m	9.00 m	90.00
3/4″ Aluminum L-Angle	7.50/m	2.50 m	18.75
5/8″ ASTM A36 Steel L-Angle	7.00/m	2.00 m	14.00
Stucco Embossed Aluminum Sheet (0.5 mm)	3.00/m^2^	1.00 m^2^	3.00
PVC-Coated Steel Cable (2.7 mm)	1.00/m	9.00 m	9.00
Final	-	-	242.95

**Table 4:** Algorithm forecast results in three different metrics.

Parameter	Temperature	Humidity	Pressure	Irradiance
MAE	1.32	6.12	0.68	76.31
RMSE	1.80	7.96	0.70	143.13
$R^{2}$	72.95%	61.05%	80.77%	78.40%

**Table 5:** RMSE of prominent work in the field of LSTM hourly weather forecasting in a 24 h horizon.

	Base Architecture	Tmp (°C)	Pr (hPa)	Rd (W/m^2^)	Hr (%)
Kreuzer et al. (2020) [22]	(ConvLSTM)	2.10	-	-	-
Hou et al. (2022) [29]	(ConvLSTM)	1.97	-	-	-
Qing and Niu (2018) [30]	(LSTM)	-	-	122.7	-
Ozbek et al. (2022) [31]	(LSTM)	-	-	-	7.51
Da Silva et al. (2024) [20]	MZDN-HF (EncDec-LSTM)	1.77	0.827	117	9.65
Current Experiment	MZDN-HF (EncDec-LSTM)	1.80	0.70	143.13	7.96

## Data Availability

All data, code, and models are openly available: Firmware: https://github.com/redstaralek/THOM-32-Firmware-P (accessed on 25 May 2025); Meteorological data: https://github.com/redstaralek/THOM-32-MeteorologicalData-2025 (accessed on 25 May 2025); MZDN-HF model: https://github.com/redstaralek/THOM-32-DATALAB/tree/018cac93ded9e3e1489a30d9fee4b2c35dd759e1 (accessed on 25 May 2025); All resources are released under open licenses to ensure reproducibility.
